# Unterstützung ärztlicher und pflegerischer Tätigkeit durch KI: Handlungsempfehlungen für eine verantwortbare Gestaltung und Nutzung

**DOI:** 10.1007/s00103-024-03918-1

**Published:** 2024-07-17

**Authors:** Tanja Bratan, Diana Schneider, Florian Funer, Nils B. Heyen, Andrea Klausen, Wenke Liedtke, Myriam Lipprandt, Sabine Salloch, Martin Langanke

**Affiliations:** 1 Competence Center Neue Technologien, Fraunhofer-Institut für System- und Innovationsforschung ISI, Breslauer Straße 48, 76139 Karlsruhe, Deutschland; 2 Institut für Ethik, Geschichte und Philosophie der Medizin, Medizinische Hochschule Hannover (MHH), Hannover, Deutschland; 3 Institut für Ethik und Geschichte der Medizin, Eberhard Karls Universität Tübingen, Tübingen, Deutschland; 4 Uniklinik RWTH Aachen, Institut für Medizinische Informatik, Rheinisch-Westfälische Technische Hochschule (RWTH) Aachen, Aachen, Deutschland; 5 Theologische Fakultät, Universität Greifswald, Greifswald, Deutschland; 6 Angewandte Ethik/Fachbereich Soziale Arbeit, Evangelische Hochschule Rheinland-Westfalen-Lippe, Bochum, Deutschland

**Keywords:** Medizin, Pflege, Künstliche Intelligenz, KI-basierte Entscheidungsunterstützungssysteme, Ethik, Medicine, Nursing, Artificial intelligence, AI-based decision support systems, Ethics

## Abstract

Klinische Entscheidungsunterstützungssysteme (*Clinical Decision Support Systems* [CDSS]), die auf *künstlicher Intelligenz* (KI) basieren, kommen als komplexe soziotechnische Innovationen zunehmend in Medizin und Pflege zum Einsatz, um die Qualität und Effizienz der Versorgung insgesamt zu verbessern und gleichzeitig begrenzten finanziellen und personellen Ressourcen zu begegnen. Neben intendierten klinischen und organisatorischen Effekten ist jedoch auch mit weitreichenden ethischen, sozialen und rechtlichen Auswirkungen KI-basierter CDSS auf Krankenversorgung und Pflege zu rechnen. Bislang sind diese normativ-sozialen Implikationen nur unzureichend erforscht. Aus dem vom BMBF geförderten Projekt DESIREE (*DEcision Support In Routine and Emergency HEalth Care: Ethical and Social Implications*) konnten Handlungsempfehlungen für eine verantwortbare Gestaltung und Nutzung klinischer Entscheidungsunterstützungssysteme abgeleitet werden. Der vorliegende Beitrag fokussiert primär auf ethische und soziale Aspekte KI-basierter CDSS, die negative Auswirkungen auf die Gesundheit von Patient:innen haben könnten. Die Handlungsempfehlungen gliedern sich in die akteursgruppenübergreifend relevanten Handlungsfelder Entwicklung, Anwendung, Aufklärung und Einwilligung, Aus‑, Fort- und Weiterbildung sowie (Begleit‑)Forschung und sind als vertiefende Ergänzungen zu existierenden Empfehlungen zu verstehen.

## Abstract (en)

Clinical decision support systems (CDSS) based on artificial intelligence (AI) are complex socio-technical innovations and are increasingly being used in medicine and nursing to improve the overall quality and efficiency of care, while also addressing limited financial and human resources. However, in addition to such intended clinical and organisational effects, far-reaching ethical, social and legal implications of AI-based CDSS on patient care and nursing are to be expected. To date, these normative-social implications have not been sufficiently investigated. The BMBF-funded project DESIREE (DEcision Support In Routine and Emergency HEalth Care: Ethical and Social Implications) has developed recommendations for the responsible design and use of clinical decision support systems. This article focuses primarily on ethical and social aspects of AI-based CDSS that could have a negative impact on patient health. Our recommendations are intended as additions to existing recommendations and are divided into the following action fields with relevance across all stakeholder groups: development, clinical use, information and consent, education and training, and (accompanying) research.

## Einleitung

Klinische Entscheidungsunterstützungssysteme (*Clinical Decision Support Systems* [CDSS]), die auf *künstlicher Intelligenz* (KI) basieren, finden zunehmend Eingang in die medizinische und auch pflegerische Versorgung [1–3]. Unter KI-basiert werden im Folgenden vornehmlich lernende Systeme verstanden (zu den verschiedenen Konzepten und Strategien von KI und ihren Potenzialen im Bereich der Gesundheitsversorgung vgl. [4–6]), wobei das Lernen kontrolliert (*Supervised*), unkontrolliert (*Unsupervised*) oder bestärkend (*Reinforced*) erfolgen kann [7]. Hinsichtlich der Art ihres Wissenserwerbs wird unterschieden zwischen *offenen* Systemen, die ihre Wissens- bzw. Datenbasis auch in der Anwendung noch eigenständig erweitern, und *geschlossenen* Systemen, deren Wissenserwerb mit Abschluss ihrer Entwicklung beendet ist. Unabhängig von der Art des Wissenserwerbs können lernende Systeme dabei entweder mit dem Zweck genutzt werden, bei bestimmten Tätigkeiten autonom zu agieren (*Decision Making*) oder als Entscheidungshilfe für humane Anwender:innen zu fungieren (*Decision Support*). Bei Letzteren reicht das Spektrum von Zweitmeinungs- und Monitoring-Tools bis hin zu Entscheidungshilfen, deren Outputs standardisiert in klinische Entscheidungsfindungsprozesse einbezogen werden und somit einen relativ hohen Verbindlichkeitsgrad haben. Besondere Herausforderungen stellen sich, wenn noch Unsicherheiten hinsichtlich der erkenntnistheoretischen Grundlagen solcher Systeme, ihrer Rolle im Versorgungsgeschehen oder hinsichtlich des Verhältnisses zwischen maschinell generierter Empfehlung und humaner Bewertung bestehen.

KI-basierte CDSS sollen dabei helfen, menschliche Schwächen im Umgang mit großen Datenmengen, Wahrscheinlichkeiten oder Zuverlässigkeit in Bezug auf Konsistenz und Genauigkeit auszugleichen und so die Versorgungsqualität zu verbessern. Mit ihnen soll die stetig zunehmende Menge an Gesundheitsdaten besser genutzt und gleichzeitig den begrenzten finanziellen und personellen Ressourcen begegnet werden. Neben solchen klinischen und organisatorischen Effekten sind weitreichende ethische, soziale und rechtliche Auswirkungen KI-basierter CDSS als komplexe soziotechnische Innovationen zu erwarten [8–14], weshalb seit einigen Jahren verschiedene Risikoklassen von KI diskutiert werden [15, 16]. Bislang sind die Implikationen solcher Systeme, bspw. bei deren routinierter Nutzung im professionellen Setting oder für die Information und Einwilligung von Patient:innen, nur unzureichend erforscht.

Das vom Bundesministerium für Bildung und Forschung (BMBF) geförderte Projekt DESIREE (*DEcision Support In Routine and Emergency HEalth Care: Ethical and Social Implications*) hat anhand von drei Fallbeispielen aus der Routineversorgung, der Akutversorgung und der Intensivpflege ethische, soziale, professionelle und technische Schlüsselaspekte der KI-basierten Entscheidungsunterstützung untersucht. Hierfür wurden zwischen April 2021 bis April 2022 insgesamt 27 Interviews mit angehenden medizinischen und pflegerischen Fachkräften und drei Fokusgruppen mit 18 Patient:innen durchgeführt. Ohne auf die empirischen Ergebnisse des Projektes im Detail eingehen zu können (vgl. [17, 18]), stellen diese eine Grundlage für die folgend dargestellten Handlungsempfehlungen dar.

Unsere Handlungsempfehlungen beziehen sich auf die im medizinethischen Kontext wohletablierten Prinzipien von Beauchamp/Childress, also jene der Autonomie, Schadensvermeidung, Fürsorge bzw. Wohltun und Gerechtigkeit [19]. Obwohl Patient:innen nicht explizit im Fokus unserer Handlungsempfehlungen stehen, ergibt sich aus diesem Prinzip, dass auch ihr Verständnis KI-basierter CDSS unterstützt werden sollte. Im Sinne des Wohltunsprinzips sollte das primäre Ziel der Nutzung KI-basierter CDSS in der Verbesserung der Behandlung liegen und beinhalten, dass die Konsequenzen zunehmender kommerzieller Interessen auf die Gesundheitsversorgung kritisch hinterfragt und möglicherweise entstehende Zielkonflikte abgefedert werden. Unter Rekurs auf Gerechtigkeit wäre zukünftig sicherzustellen, dass KI-basierte CDSS weder als Systeme erster noch zweiter Klasse verwendet werden und bei nachgewiesenem Nutzen alle Patient:innen profitieren können, ohne dass dadurch sinnvolle menschliche Interaktionen ersetzt werden.

Unsere Handlungsempfehlungen adressieren vor allem den Umgang mit KI-basierten CDSS vor ihrer Etablierung in der Routineversorgung und richten sich daher an Entwickler:innen und Hersteller:innen, Anwender:innen KI-basierter CDSS und das medizinische und pflegerische Bildungssystem. Sie gliedern sich in die akteursgruppenübergreifend relevanten Handlungsfelder Entwicklung, Anwendung, Aufklärung und Einwilligung, Aus‑, Fort- und Weiterbildung sowie (Begleit‑)Forschung. Die Empfehlungen wurden durch einschlägige Expert:innen kommentiert und diskutiert (s. Danksagung). Der Fokus liegt primär auf ethischen und sozialen Aspekten KI-basierter CDSS, die negative Auswirkungen auf die Gesundheit von Patient:innen haben können (bspw. Systeme, die Diagnose- oder Therapieempfehlungen geben).

Die vorliegenden Empfehlungen erheben keinen Anspruch darauf, das Handlungsfeld vollständig abzudecken oder in Konkurrenz zu Empfehlungen von Fachgesellschaften und anderen Gremien [11, 20, 21] zu treten. Vielmehr adressieren sie Aspekte, zu denen im Rahmen des DESIREE-Projekts gezielt, interdisziplinär und eigenständig geforscht wurde, und sind als vertiefende Ergänzungen zu existierenden Empfehlungen zu verstehen.

## Handlungsfeld Entwicklung

Werden KI-basierte CDSS für Diagnostik oder Therapie eingesetzt, fallen sie innerhalb der EU unter die Medizinprodukteverordnung [22]. In der Entwicklung eines KI-basierten CDSS muss daher stets der Nachweis erbracht werden, dass es entsprechend seiner Risikoklasse den Anforderungen dieses EU-Gesetzes entspricht. Hierbei stehen das Risikomanagement zur Einhaltung der Patient:innensicherheit, der Nachweis der angestrebten Leistung und die regulatorischen Anforderungen im Vordergrund. Dazu gehören insbesondere Datenschutz, Datensicherheit, Zuverlässigkeit und Genauigkeit sowie der Nachweis der Erfüllung grundlegender Sicherheits- und Leistungsanforderungen. Zudem erfordern die regulatorischen Rahmenbedingungen eine klinische Bewertung. Liegen keine klinischen Daten vor, müssen Hersteller:innen diese durch klinische Prüfung(en) generieren. Sie müssen nachweisen, dass ihre Systeme dem Stand der Technik entsprechen und hinsichtlich Leistung und Sicherheit dem Goldstandard nicht unterlegen sind [15].

Darüber hinaus reguliert das kürzlich verabschiedete Gesetz über künstliche Intelligenz der Europäischen Kommission (*AI Act*) KI-Systeme entsprechend ihres erwartbaren Risikos (*Unacceptable Risk, High Risk, None of Both;* [15, 16]). Bislang kommen im Bereich von Medizin und Pflege primär geschlossene KI-basierte CDSS in die Routineversorgung. Sollten künftig offene Systeme oder Systeme, die sich verändern, eingesetzt werden, bedürfte es nach aktueller Gesetzeslage einer neuen Konformitätsbewertung.

Die folgenden Empfehlungen beruhen auf Untersuchungen zur einschlägigen Regulatorik. Sie lehnen sich besonders an die Handreichung *Good Machine Learning Practice for Medical Device Development* der U.S. Food and Drug Administration (FDA; [23]) an. Dabei richten sie den Fokus auf Aspekte, die zwar im Prinzip regulatorisch geregelt sind, aber im Kontext der Entwicklung KI-basierter CDSS eine neue Relevanz und Dringlichkeit aufweisen:

- Aufgrund der Komplexität und Tragweite der Herausforderungen, die mit der Einbindung KI-basierter CDSS in Medizin und Pflege verbunden sein werden, sollte die Entwicklung solcher Systeme von interdisziplinären Teams über den gesamten Produktlebenszyklus hinweg begleitet werden. Neben den Entwickler:innen gehören dazu auch Mediziner:innen sowie Expert:innen für Datenschutz und Ethik. Die im Team erforderlichen Kompetenzen umfassen die Sicherstellung der Datenqualität, das Datenmanagement, die Datenintegrität, die Cybersicherheit sowie die Methodik des Risikomanagements.
- Nach Möglichkeit sollten KI-basierte CDSS partizipativ und kokreativ, d. h. unter aktiver Einbeziehung von Stakeholder:innen (insbesondere künftigen Anwender:innen und Patient:innen), entwickelt werden. Es sollte von der Möglichkeit Gebrauch gemacht werden, durch etablierte Verfahren der integrativen Modellierung mögliche Schwächen von Menschen bzw. KI-basierter CDSS bereits in der Entwicklungsphase zu erkennen und auszugleichen sowie vorhandene Potenziale positiv zu verstärken.
- Die Diversität in den relevanten Patient:innenpopulationen, z. B. hinsichtlich Alter, Geschlecht, Ethnie oder Phänotyp, ist in den Trainings- und Testdatensätzen möglichst gut abzubilden, um eine Diskriminierung durch eine unzureichende, naturgemäß fragmentierte Datenbasis zu vermeiden. Etwaige Einschränkungen in der Datenbasis sollten den Zielgruppen, die von der Anwendung erwiesenermaßen besonders profitieren, sowie den Gruppen, für die ein Nutzen nicht gesichert oder fraglich ist, klar benannt werden.
- Obwohl zu erwarten ist, dass Praxisrelevanz und Qualität der Outputs KI-basierter CDSS für unterschiedliche Patient:innengruppen und Krankheitsbilder variieren werden, sollte ein *Off-Label Use* KI-gestützter Systeme für andere Patient:innengruppen bzw. Krankheitsbilder als die ursprünglich intendierten unter entsprechender Abwägung und systematischer Evaluation grundsätzlich möglich sein, um potenziellen Nutzen auch für neue Zielgruppen zu realisieren.
- Die Prüfung von KI-basierten CDSS erfordert formative und summative Usability-Tests. Diese Prüfungen sollten unter realitätsnahen klinischen Bedingungen und unter Einbeziehung zukünftiger Anwender:innengruppen erfolgen. Ziel muss es sein, den Nutzungskontext mit allen relevanten Umgebungsvariablen (z. B. Versorgungsprozess, Teaminteraktion, Stress und Akustik) zu simulieren, um die Gebrauchstauglichkeit zu überprüfen und mögliche Anwendungsfehler zu ermitteln, um Patient:innenschäden zu vermeiden. Dies impliziert den Einsatz realitätsnaher Anwendungsfälle (*Use Cases*).
- Bei der Entwicklung ist zu bedenken, dass die Nutzung KI-basierter CDSS nicht zum Verlust von Wissen, Fähigkeiten und Kompetenzen (*De-Skilling*) bei zukünftigen Nutzer:innen führen darf. De-Skilling könnte dadurch vermieden werden, dass inhaltliche Begründungen für Empfehlungen KI-basierter CDSS so weit wie möglich transparent und nachvollziehbar gemacht werden. Die Vermeidung des Risikos von De-Skilling muss ebenfalls organisatorisch in den Blick genommen werden, bspw. in der Ausbildung oder durch berufsbegleitende Fortbildungen und Trainings.

## Handlungsfeld Anwendung in der Versorgung

KI-basierte CDSS finden zunehmend Eingang in medizinische Versorgung und Pflege [2, 3]. Vielversprechende Anwendungsbeispiele sind radiologische Assistenzsysteme zur Interpretation von Daten aus bildgebenden Verfahren (v. a. CT, MRT, Röntgen; [24–26]) sowie bestimmte Systeme zum verbesserten Management chronischer [1] oder onkologischer Erkrankungen [27]. Jedoch fehlt es für viele Anwendungsfelder noch an Beispielen guter Praxis, aus denen Kriterien für eine gelingende Integration dieser neuen Technologie in die Routineabläufe von Medizin und Pflege ableitbar wären.

Für medizinische Versorgung und Pflege zeichnen sich 2 Herausforderungen ab: Erstens bestehen große Unsicherheiten hinsichtlich der künftigen Rollen- und Funktionsverteilung in einem Versorgungsgeschehen, das von der Nutzung KI-basierter CDSS geprägt ist. Es gibt Unsicherheiten hinsichtlich der Zuständigkeiten und Verantwortlichkeiten (vgl. [18]), insbesondere im Umgang mit Empfehlungen KI-basierter CDSS, sowie deren Verbindlichkeit bzw. der verbleibenden Ermessensspielräume der Anwender:innen. Zweitens sind die Auswirkungen KI-basierter CDSS auf die Beziehung zwischen den Ärzt:innen bzw. Pflegekräften und ihren Patient:innen derzeit weder in Art noch Qualität ausreichend vorhersehbar; in vielerlei Weise ist noch unklar, wie mit möglichen unerwünschten Auswirkungen des Einsatzes KI-basierter CDSS auf diese Beziehung in Versorgung und Pflege umzugehen ist [8–14, 18].

Vor diesem Hintergrund wurden auf Basis der DESIREE-Projektergebnisse folgende Empfehlungen erarbeitet:

- Die Grenze zwischen KI-gestütztem *Decision Making* und *Decision Support* muss in der Anwendung scharf gezogen werden. Insbesondere Fachgesellschaften kommt es zu, Status und Rolle KI-basierter CDSS für Versorgungskontexte unter Einbezug transdisziplinärer Expertise spezifisch zu definieren und legitime von illegitimen Einsatzoptionen zu unterscheiden. Im Sinne geteilter Entscheidungsfindung sollte die Entscheidungshoheit über konkrete Therapie- oder Pflegeoptionen bei Ärzt:innen bzw. Pfleger:innen und den Patient:innen selbst verbleiben. Die Besonderheiten des Einzelfalls [28], individuelle Bedürfnisse und Belange von Patient:innen, Kontextsensitivität und Zurechenbarkeit von medizinischen und pflegerischen Entscheidungen sind im Sinne der Standards evidenzbasierter Medizin zu gewährleisten und ein neuer *Computer-Paternalismus* zu vermeiden. Die Patient:innen-Autonomie ist zu stärken.
- Ein Recht auf begründetes Befolgen bzw. Nichtbefolgen KI-generierter Empfehlungen durch Ärzt:innen, Pflegekräfte und/oder Patient:innen sollte bestehen bleiben. Angesichts der Risiken einer unkritischen Übernahme von CDSS-Empfehlungen (*Automation Bias bzw. Overreliance)* sollte die konkrete Ausgestaltung eines solchen Rechts oder die Notwendigkeit besonderer Begründungspflichten durch die zuständigen Fachgesellschaften definiert werden, um betroffenen Berufsgruppen nötige Handlungssicherheit zu geben. Es sind individuelle und kollektiv geteilte rechtliche und ethische Verantwortlichkeiten sowie Ermessensspielräume zu definieren, um eine Verantwortungsdiffusion zu vermeiden.
- Die Anwender:innen müssen essenzielle Informationen zu Art, Umfang und Qualität der Trainingsdatenbasis, zur Zweckbestimmung des Systems, seiner Eignung bzw. Nichteignung für spezifische Subgruppen sowie zu seinen Limitationen erhalten. Dies kann in Schulungen oder durch Bereitstellung von zielgruppenadaptierten Informationsmaterialien geschehen.
- Auch wenn *offene* selbstlernende Systeme nach der Medizinprodukteverordnung in ihrer derzeit geltenden Form [22] wohl keine zulassungsfähigen Medizinprodukte darstellen, erscheint es geboten, dass bei einer künftigen Nutzung solcher Systeme den Anwender:innen prinzipiell wünschenswerte Verbesserungen der Systeme durch das Hinzufügen von neuen Realweltdaten oder von Parametern anderer Modelle kommuniziert werden. Die Nutzung von Feedbacksystemen, über die den Entwickler:innen/Hersteller:innen Bedenken oder Fehler aus der Benutzung KI-basierter CDSS heraus mitgeteilt werden können, sollte in der Praxis fest verankert und gefördert werden.
- KI-basierte CDSS sollten zukünftig so eingesetzt werden, dass die interpersonelle Beziehungsebene intakt bleibt, d. h., dass sowohl das Vertrauensverhältnis zwischen Patient:innen und ihren Ärzt:innen bzw. Pfleger:innen als auch die Beziehung zwischen Ärzt:innen bzw. Pfleger:innen und deren Arbeitgeber:innen keinen Schaden nimmt.

## Handlungsfeld Aufklärung und Einwilligung

Die Aufklärung über und die Einwilligung in medizinische Maßnahmen gelten als unverzichtbarer medizinethischer und -rechtlicher Standard. Damit einwilligungsfähige Patient:innen eine informierte Einwilligung (*Informed Consent*) geben können, bedarf es der ärztlichen Aufklärung über Art, Bedeutung und Tragweite sowie über mögliche Alternativen zu vorgeschlagenen Maßnahmen [29, 30]. Aufgrund der Komplexität und Neuartigkeit KI-gestützter Systeme bestehen besondere Anforderungen an die informierte Einwilligung. Dies gilt umso mehr, da die Patient:innen laut unserer Erhebungen typische Bedenken wie (a) Überwachungsrisiken, (b) Stigmatisierungsrisiken bei Datenschutzverstößen, (c) Risiken durch Normierung und Standardisierung, (d) mögliche Loyalitätskonflikte mit behandelnden Ärzt:innen sowie (e) Risiken im Zusammenhang mit einer fehlenden Berücksichtigung der individuellen Krankheitsgeschichte und der Präferenzen von Patient:innen beim Einsatz KI-basierter Systeme äußern [18].

Hinsichtlich des angemessenen Umfangs einer Aufklärung zur Nutzung KI-basierter CDSS wurden im Projekt vielfältige Unsicherheiten und Diskrepanzen zwischen den Stakeholder:innen festgestellt. Die Aufzeichnung und Auswertung umfangreicher Patient:innendaten wirft Fragen auf, welche Kompetenzen und Kenntnisse bei Patient:innen vorausgesetzt werden können und welche bei Bedarf im Aufklärungsgespräch vermittelt werden müssen. Nach Einschätzung der befragten Stakeholder:innen erfordert die Nutzung datenintensiver Verfahren im medizinischen Kontext eine Ausweitung der Aufklärung auf Themen der Datensicherheit, des Datenschutzes oder der Funktionsweise von Algorithmen zur Datenauswertung. Dabei ist zu berücksichtigen, dass der Aufklärungsbedarf beim frühen Einsatz medizinischer Innovationen typischerweise hoch ist, während er mit zunehmender Etablierung einer neuen Technik erwartbar zurückgeht. Dessen unbeschadet ist davon auszugehen, dass bei der Nutzung KI-basierter CDSS aufgrund ihrer teils weitreichenden Einflüsse auf die Versorgung und ihrer medialen Präsenz ein grundsätzlicher Aufklärungsbedarf über längere Zeit bestehen bleiben wird.

Vor dem skizzierten Hintergrund hat das DESIREE-Konsortium folgende Empfehlungen erarbeitet:

- Patient:innen sollten standardmäßig darüber informiert werden, wenn im Rahmen ihrer Behandlung ein KI-basiertes CDSS verwendet wird, das bislang noch nicht in der Versorgung etabliert ist und relevante Auswirkungen auf sie haben kann. Sofern Alternativen existieren, sind diese neutral und gleichberechtigt zu präsentieren.
- Es besteht Klärungsbedarf, wie, in welcher Weise und von wem über Datensicherheit und Datenschutz aufzuklären ist. Sofern relevant, sollte dabei auch über eine mögliche Sekundärnutzung von Daten aufgeklärt werden.
- Typische Bedenken von Patient:innen, bspw. hinsichtlich potenzieller Diskriminierungen oder Auswirkungen auf das Behandlungsverhältnis, sind bedarfs- und situationsabhängig zu adressieren. Um eine informierte Einwilligung zu gewährleisten, muss grundsätzlich das Daten- und Technikverständnis individuell und patient:innenbezogen berücksichtigt werden.
- Im Aufklärungsgespräch sollten Ärzt:innen transparent und nachvollziehbar auf die begrenzte Aussagekraft von Ergebnissen hinweisen, die mithilfe KI-basierter CDSS generiert wurden, insbesondere mit Blick auf die gemeinsame Entscheidungsfindung.
- Ähnlich wie bei anderen Maßnahmen in der medizinischen Praxis sollten Patient:innen die Option haben, die Nutzung eines KI-basierten CDSS abzulehnen, soweit dieses im klinischen Workflow als spezifisches Prozessglied abgrenzbar und identifizierbar ist. Eine einmal gegebene Einwilligung muss zu einem späteren Zeitpunkt widerrufbar sein.
- Um Handlungssicherheit zu unterstützen, erscheint es sinnvoll, das Aufklärungs- und Einwilligungsverfahren bei Nutzung KI-basierter CDSS (etwa durch unterstützende Aufklärungsmerkblätter, Filme und geeignete digitale Angebote) zu standardisieren.

## Handlungsfeld Aus‑, Fort- und Weiterbildung

Nutzende KI-basierter CDSS werden, wie bei Medizinprodukten üblich, nach § 4 der Medizinprodukte-Betreiberverordnung über die vom Hersteller definierte Funktionsweise und Leistungsfähigkeit der Systeme, einschließlich ihrer Grenzen, eingewiesen [31]. KI-basierte CDSS, die zur Diagnose oder Therapieempfehlungen genutzt werden und damit negative Auswirkung auf die Gesundheit von Patient:innen haben könnten, unterscheiden sich jedoch in wesentlichen Zügen von Medizinprodukten ohne KI und erfordern daher für eine informierte Nutzung ein schematisches Grundverständnis über die Funktionsweise von KI. Die dafür notwendige Datenkompetenz (*Data Literacy*) ist in den betroffenen Berufsgruppen jedoch unterschiedlich ausgeprägt [32] und wird in den jeweils relevanten Ausbildungscurricula unterschiedlich umgesetzt [33, 34].

Sofern Ärzt:innen und Fachpflegende die rechtliche und moralische Letztverantwortung für das eigene medizinische bzw. pflegerische Handeln tragen sollen, sind hierfür geeignete Befähigungsmaßnahmen erforderlich. Diese umfassen eine ausreichende Kompetenzbildung zur Beurteilung KI-generierter CDSS-Empfehlungen sowie eine ausreichende Bereitstellung von Informationen über die Qualität und Sicherheit dieser Outputs. Bei zukünftigen Anwender:innen bestehen laut unserer Erhebungen jedoch erhebliche Unsicherheiten im Verständnis von CDSS sowie hinsichtlich ihrer Benutzung und in Bezug auf ethische, rechtliche und weitere Aspekte. Auch die Entwickler:innen thematisierten eigene Unsicherheiten in Bezug auf ethische und soziale Aspekte KI-basierter CDSS.

Vor diesem Hintergrund stellt das DESIREE-Projekt folgende Empfehlungen zur Diskussion:

- Eine interdisziplinäre Verständigung über erforderliche Kompetenzen von Ärzt:innen und Fachpflegenden (bzgl. Daten- und Technikverständnis, kommunikativer Kompetenzen zur Aufklärung) ist erforderlich. Es gilt zu prüfen, wer die Federführung einer solchen Verständigung übernehmen könnte.
- Ausbildungscurricula in Medizin und Pflege sollten unter Berücksichtigung des jeweilig erwartbaren Kompetenz- und Erfahrungsstands in Bezug auf Funktionsweisen sowie Möglichkeiten und Grenzen KI-basierter Systeme erweitert werden. Die Ausbildung sollte zu Erklärungen gegenüber Patient:innen befähigen (vgl. Handlungsfeld Aufklärung und Einwilligung).
- Bei der Einweisung in Funktion und Leistungsfähigkeit KI-basierter CDSS gilt es sicherzustellen, dass Ausfallszenarien konzeptionell eingeschlossen sind. In den nutzenden Organisationen ist Klarheit über niederschwellige Kommunikationswege für die Fehlerkommunikation zu schaffen, sodass Vorkommnisse zeitnah dem Bundesinstitut für Arzneimittel und Medizinprodukte (BfArM) gemeldet werden.
- Es besteht Bedarf an Weiterbildungsangeboten in Medizin und Pflege für berufstätige Personen. Hierfür könnte z. B. das bestehende Angebot des KI-Campus ausgeweitet und von weiteren Landesärztekammern unterstützt werden. Ähnliche Angebote sollten auch für Fachpflegende etabliert werden.
- Der Einsatz KI-basierter CDSS ermöglicht grundsätzlich eine individuelle Qualitätskontrolle. Etwaige Defizite, bspw. klinischer oder pflegerischer Kompetenzen oder in der Nutzung des CDSS, die durch KI-basierte CDSS identifiziert wurden, sollten konstruktiv über Schulungen adressiert werden, welche die Fähigkeit zur selbstständigen Plausibilitätsprüfung fördern.
- Auch wenn KI-basierte CDSS zukünftig bestimmte klinische und pflegerische Aufgaben regulär unterstützen können, bleibt die Aus- und Weiterbildung in medizinischen und pflegerischen Kernkompetenzen erforderlich, um die Versorgungssicherheit zu gewährleisten und De-Skilling zu vermeiden (vgl. Handlungsfeld Entwicklung).
- Empfehlungen KI-basierter CDSS sollten in Versorgungsteams besprochen werden, um die Anwendungspraxis kontinuierlich zu reflektieren. Hierfür können Fallkonferenzen oder Fallbesprechungen geeignete Formate darstellen. Klinische Ethikkomitees sind dabei einzubinden.
- Es sollten Maßnahmen zur Sensibilisierung und Weiterbildung von Entwickler:innen bezüglich ethischer und sozialer Aspekte von CDSS eingeführt werden, damit das Wissen künftig stärker in die Entwicklung einfließen kann. Dies kann durch gezielte Schulungsangebote oder die Ausweitung von Dialogformaten zwischen Entwickler:innen und Expert:innen aus dem Bereich der ELSI-Fächer (*Ethical, Legal and Social Implications*) geschehen.

## Handlungsfeld (Begleit‑)Forschung zu CDSS und Evaluation

Die aktuelle Forschung zu KI-basierten CDSS ist oft wenig interdisziplinär. In der technischen Forschung werden ethische, rechtliche und sozialwissenschaftliche Implikationen (ELSI) wenig berücksichtigt [3] und die ELSI-Forschung zu KI-basierten CDSS bewegt sich in unzureichender Nähe zur Technikentwicklung. Trotz der intensiven, wissenschaftlichen sowie öffentlichen Debatte zu KI-basierten CDSS in den letzten Jahren und zahlreicher Leitlinien, *White Papers* und Übersichtsarbeiten sind insbesondere die Effekte des Einsatzes KI-basierter CDSS in der Versorgungspraxis nach wie vor nur unzureichend erforscht [1, 27]. Dabei steht außer Frage, dass KI-basierte CDSS weitreichende Wirkungen auf die Versorgung haben werden. Dennoch ist bislang weder geklärt, wie diese Systeme in die Entscheidungsprozesse eingebunden werden [12], noch, welche Auswirkungen dies auf die Versorger:innen-Patient:innen-Beziehung haben wird [10]. Vor diesem Hintergrund lassen sich folgende Empfehlungen formulieren:

- Auch nach der Zulassung und den dafür notwendigen klinischen Studien (vgl. Handlungsfeld Entwicklung) sollte die Wirksamkeit KI-basierter CDSS routinemäßig in der regulären Versorgung und unter realen Bedingungen bspw. auf Superiorität geprüft werden (Phase-IV-Studien/Versorgungsforschung). Die Studienergebnisse sollten über die wissenschaftliche Gemeinschaft hinaus in den Bereichen Versorgung und Entwicklung verbreitet werden, damit sie dort berücksichtigt werden können.
- Mit zunehmender Verbreitung KI-basierter CDSS können mehrere Systeme in einem Versorgungssetting gleichzeitig Anwendung finden [35], sodass – wo relevant – kumulierte Effekte mehrerer KI-basierter CDSS zu berücksichtigen sind und ihr Zusammenspiel in konkreten Versorgungssituationen zu untersuchen ist.
- Es sind Studien notwendig, in denen der Einsatz KI-basierter CDSS in der Praxis untersucht wird. Dazu gehören bspw. Auswirkungen auf das Behandlungsverhältnis in Versorgung und Pflege, klinische sowie partizipative Entscheidungsprozesse, die Verwendung zeitlicher Ressourcen oder möglicherweise auftretende klinische Kompetenzverluste (De-Skilling).
- Es bedarf Forschung zum übersteigerten Vertrauen in KI-basierte CDSS und zu daraus möglicherweise resultierenden Fehlern (*Automation Bias* und *Automation Complacency*), und wie derartige Verzerrungen zukünftig vorbeugend adressiert werden können.
- Es ist davon auszugehen, dass in Zukunft auch KI-basierte CDSS in die Routineversorgung kommen, die kontrolliert offen maschinelles Lernen nutzen. Dadurch ergibt sich schon jetzt Forschungsbedarf zu den klinischen, ethischen, rechtlichen, sozialen und ökonomischen Potenzialen und Risiken solcher Systeme im Rahmen unabhängiger Begleitforschung.
- Es besteht weiterer Forschungsbedarf zu den Aufklärungswünschen und -bedarfen der Patient:innen hinsichtlich der Funktionsweisen KI-basierter CDSS (vgl. Handlungsfeld Aufklärung und Einwilligung). Dabei ist ein angemessener und sinnvoller Detaillierungsgrad der Aufklärung zu bestimmen.

## Schlussfolgerungen

Die vorliegenden Empfehlungen werden zu einem Zeitpunkt präsentiert, zu dem für die Nutzung KI-basierter CDSS in Medizin und Pflege zwar bereits erste erfolgversprechende Beispiele guter Praxis vorliegen, jedoch binnen- und interdisziplinäre, berufsrollenbezogene oder gar gesamtgesellschaftliche Effekte nach Art und Ausmaß kaum abzusehen sind. Sie können künftigen Herausforderungen möglicherweise nicht oder nur ansatzweise Rechnung tragen, bspw. hinsichtlich Fragen, die sich aus der routinierten Nutzung offener lernender Systeme zum Zweck der Entscheidungsunterstützung oder -findung ergeben. Dennoch verweisen sie auf Notwendigkeiten, die sich bereits jetzt abzeichnen, um eine verantwortbare Gestaltung und Nutzung aktueller und zukünftiger KI-basierter CDSS zu ermöglichen.

Angesichts erheblicher Unsicherheiten hinsichtlich der Effekte einer fortschreitenden Nutzung KI-basierter CDSS in Medizin und Pflege ist den organisationalen, sozialwissenschaftlichen und ethischen Aspekten dieses Innovations- und Transformationsgeschehens noch mehr Aufmerksamkeit als bisher zu schenken. Dies gilt sowohl in der ärztlichen und pflegerischen Ausbildung als auch in der reflexiven Begleitung durch die spezifischen professionellen Fachgesellschaften sowie in den betroffenen Forschungszweigen und Disziplinen.
